# Early Follow‐Up of Arthroscopic Latarjet Procedure with Screw or Suture‐Button Fixation for Recurrent Anterior Shoulder Instability

**DOI:** 10.1111/os.12781

**Published:** 2020-11-16

**Authors:** Yi Wang, Zhi‐you Zhou, Yong‐jin Zhang, Chong‐ru He, Chen‐chen Xue, Wei‐dong Xu, Zi‐min Wang

**Affiliations:** ^1^ Department of Orthopaedic Surgery Third Affiliated Hospital of Navy Medical University Shanghai China; ^2^ Department of Orthopaedic Surgery First Affiliated Hospital of Navy Medical University Shanghai China

**Keywords:** Arthroscopy, Instability, Latarjet, Shoulder

## Abstract

**Objective:**

To evaluate the early clinical and radiographic results of arthroscopic Latarjet procedure using screw or suture‐button fixation in patients with recurrent anterior shoulder dislocation.

**Methods:**

Twelve patients who underwent arthroscopic Latarjet procedure between January 2015 and December 2018 at our institution were retrospectively studied. Data of the patients' history, including age, gender, side of affected arm, body mass index (BMI), and the number of dislocations since fist dislocation were collected. Preoperative and postoperative clinical follow‐up data were evaluated using Walch–Duplay score, American Shoulder and Elbow Society (ASES) score, and modified Rowe score. Active external rotation and active internal rotation at 90° of abduction as well as active elevation were evaluated preoperatively and postoperatively. The position and healing condition of the transferred coracoid bony graft were also assessed using computed tomography (CT) and Mimics 19.0 software.

**Results:**

Mean follow‐up was 24.9 months (range, 13 to 53 months) of all patients. At final follow‐up, the average ASES score (preoperative *vs* postoperative values) had improved from 68.9 ± 7.9 to 91.1 ± 6.1 in screw fixation group and 68.9 ± 8.9 to 87.5 ± 6.7 in suture‐button fixation group; the average Rowe score (preoperative *vs* postoperative values) had improved from 25.0 ± 8.4 to 92.5 ± 4.2 in screw fixation group and 21.7 ± 13.7 to 93.3 ± 4.1 in suture‐button fixation group; the average of Walch–Duplay score (preoperative *vs* postoperative values) had improved from 12.5 ± 15.1 to 91.7 ± 4.1 in screw fixation group and 18.3 ± 20.7 to 88.3 ± 7.5 in button fixation group. The forward flexion was 175.0° ± 8.4° preoperatively and 178.3° ± 4.1° postoperatively in screw fixation group while 174.8° ± 10.2° preoperatively and 175.0° ± 5.5° postoperatively in suture‐button fixation group. The active external rotation was 77.5° ± 5.2° preoperatively and 71.7° ± 4.1° postoperatively in screw fixation group while 72.5° ± 6.9° preoperatively and 68.3° ± 7.5° postoperatively in suture‐button fixation group. The average of active internal rotation was 66.7° ± 6.1° preoperatively and 67.5° ± 6.1° postoperatively in screw fixation group while 68.3° ± 11.3° preoperatively and 66.7° ± 7.5° postoperatively in suture‐button fixation group. In postoperative CT scan, 91.7% grafts midline center were located at or under the equator in the en face view; 75% of the bone blocks were flush to the glenoid face in the axial view, with only two grafts exhibiting slight medial placement in screw fixation group (33.3%) and one graft exhibiting slight lateral placement in suture‐button fixation group (16.7%). All grafts achieved bone union. Graft absorption mostly occurred outside of the “best‐fit” circle. The average bony absorption rates of the coracoid grafts were 25.2% and 10.18% in screw fixation group and suture‐button fixation group, respectively, at 6 months postoperative follow‐up.

**Conclusion:**

Both suture‐button fixation and screw fixation techniques in arthroscopic Latarjet procedure revealed excellent clinical outcomes with low complication rates in the early follow‐up. The suture‐button fixation exhibited a flexible fixation pattern that allowed for self‐correction to some extent, even slight lateralization could finally remodel over time.

## Graphical abstract

The Latarjet prodedure is becoming increasingly popular when considering treating severe anterior shoulder instability. Whether operating openly or arthroscopily, the coracoid fixation method varies among surgeons and arouse unsolved disputes in recent years. Arthroscopic Latarjet procedure is a challenging yet viable technique, achieving equal results to the open technique with advantages of the arthroscopic setting.

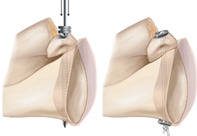

## Introduction

Recurrent dislocation of the shoulder joint may cause severe anterior shoulder instability and needs to be treated properly^1^. Anterior glenohumeral instability has an incidence of 0.08 per 1000 persons in the general American population and is typically caused by a traumatic injury^2^. Instability results from a variety of complex factors including soft tissue pathology such as a labral tear, glenoid or humeral head bone loss, and ligamentous laxity. An anteroinferior glenoid rim injury, which is often referred to as a Bankart lesion, is the most common injury after an anterior shoulder dislocation^3^. Bony injuries of the humerus referred as a Hill–Sachs lesion occurs approximately 40%–90% in first time dislocation and in up to 100% of patients experiencing recurrent instability^4^. Injuries of glenoid bone have been reported in 22% of initial dislocations and in as many as 90% of patients with recurrent instability^5^, ^6^. In traumatic anterior dislocation, co‐injury of the glenoid Bankart lesion and humerus Hill–Sachs lesion is often detectable^7^. Loss of bone on the glenoid decreases the contact area with humeral head available and leads to recurrent instability.

The Bankart repair was commonly used to treat patients with recurrent instability of the shoulder in the past, but more surgeons are now turning to a Latarjet procedure as their first choice for young and active patients with a higher risk of recurrent instability.

The Latarjet procedure is usually recommended when there is a significant bone loss of more than 20%–25%. It has been proven as an effective method to treat this defective glenoid since it was proposed by French surgeon Latarjet in 1954^8^, ^9^, ^10^. The Latarjet procedure is a surgical technique that is aimed at restoring the congruity of the glenohumeral joint using a section of coracoid process as an augmentation of the anteroinferior glenoid rim. With the improvement of arthroscopic techniques, the arthroscopic Latarjet procedure is becoming increasingly popular^11^, ^12^, ^13^, ^14^. The Latarjet procedure is a reliable method with good results for the treatment of anterior instability. Hovelius *et al*. reported a better result in Latarjet procedure with a significantly lower rate of recurrence and higher clinical scores compared with Bankart repair^15^. Zhu *et al*. found notably less graft resorption and similar postoperative scores for patients in the arthroscopic Latarjet group compared with patients in the open Latarjet group^16^.

Despite the satisfying results of Latarjet procedure, a meta‐analysis showed that overall complication rates were 23.7% in arthroscopic Latarjet and 15.3% in open Latarjet. Among them, the recurrence rates were 6.5% and revision surgery rates were 5.7%^17^. Many surgeons believe a better fixation method of coracoid may reduce the recurrence and revision rates. Whether operating openly or arthroscopically, the coracoid fixation method varies among surgeons and has incited unsolved disputes in recent years^18^. Laffose *et al*.^11^ described a procedure in which the screw fixation of the coracoid was similar to the original Latarjet technique. However, a significant proportion of complications are related to screw fixation including impingement with soft tissue, screw bending or pullout, bone‐block fractures or nonunion, graft resorption, and others^19^, ^20^. Alviet *et al*. compared stainless steel cortical screws with partially threaded cancellous screws in fresh‐frozen bodies and found no statistically significant difference in energy or cycles to failure^21^.

In a biomechanical study, Massin *et al*. compared five different fixation systems including two malleolar screws, one screw with washer, two 3.5‐mm self‐compressive screws, one 4‐mm self‐compressive screw associated with one 3‐mm self‐compressive screw, and endo‐button. They found a comparable biomechanical resistance between these fixation systems except a single screw^22^. Boileau *et al*.^13^ proposed an innovative suture‐button fixation. They evaluated the clinical outcomes with suture‐button fixation and consider it as an alternative to screw fixation for Latarjet procedure^23^. Similar to suture‐button fixation for distal tibiofibular syndesmosis, this suture‐button fixation might be considered an elastic fixation allowing minimal motion of the bone healing area^24^. This method might help avoid impingement of the humeral head caused by malposition of the coracoid bony graft or its screws above the glenoid level with no reported neurological complications. Also, Provencher *et al*. found a comparable biomechanical strength for coracoid bone block fixation between the screw and suture‐button fixation techniques in Lataejet procedure^25^. The procedure remains technique demanding and, to our knowledge, few centers have reported clinical and radiologic outcomes for this surgical technique in China^26^.

The purpose of this study is to: (i) assess the early clinical results of suture‐button fixation technique during the arthroscopic Latarjet procedure; (ii) evaluate remodeling and healing of the transferred coracoid bony grafts using suture‐button fixation compared with screw fixation; (iii) explore the factors influencing the effects of different fixation methods in Latarjet procedure. We hypothesized that suture‐button fixation would promote bony graft healing and remodeling, and could compete with screw fixation in postoperative clinical outcomes.

## Patients and Methods

### *Ethics Approval*

Approval of this retrospective study was obtained from the Changhai Hospital ethical committee (No.168, Changhai Road, Yangpu District, Shanghai, China). All patients gave informed consent before surgical procedures.

### *Patient Data*

Inclusion criteria: (i) patients with recurrent anterior shoulder instability; (ii) presence of anterior glenoid bone defect >20% or Instability Severity Index Score > 6; (iii) treatment with a modified arthroscopic Latarjet technique; (iv) fixed with screws or suture‐buttons; (v) evaluated with a minimum follow‐up of 12 months.

**Fig 1:**
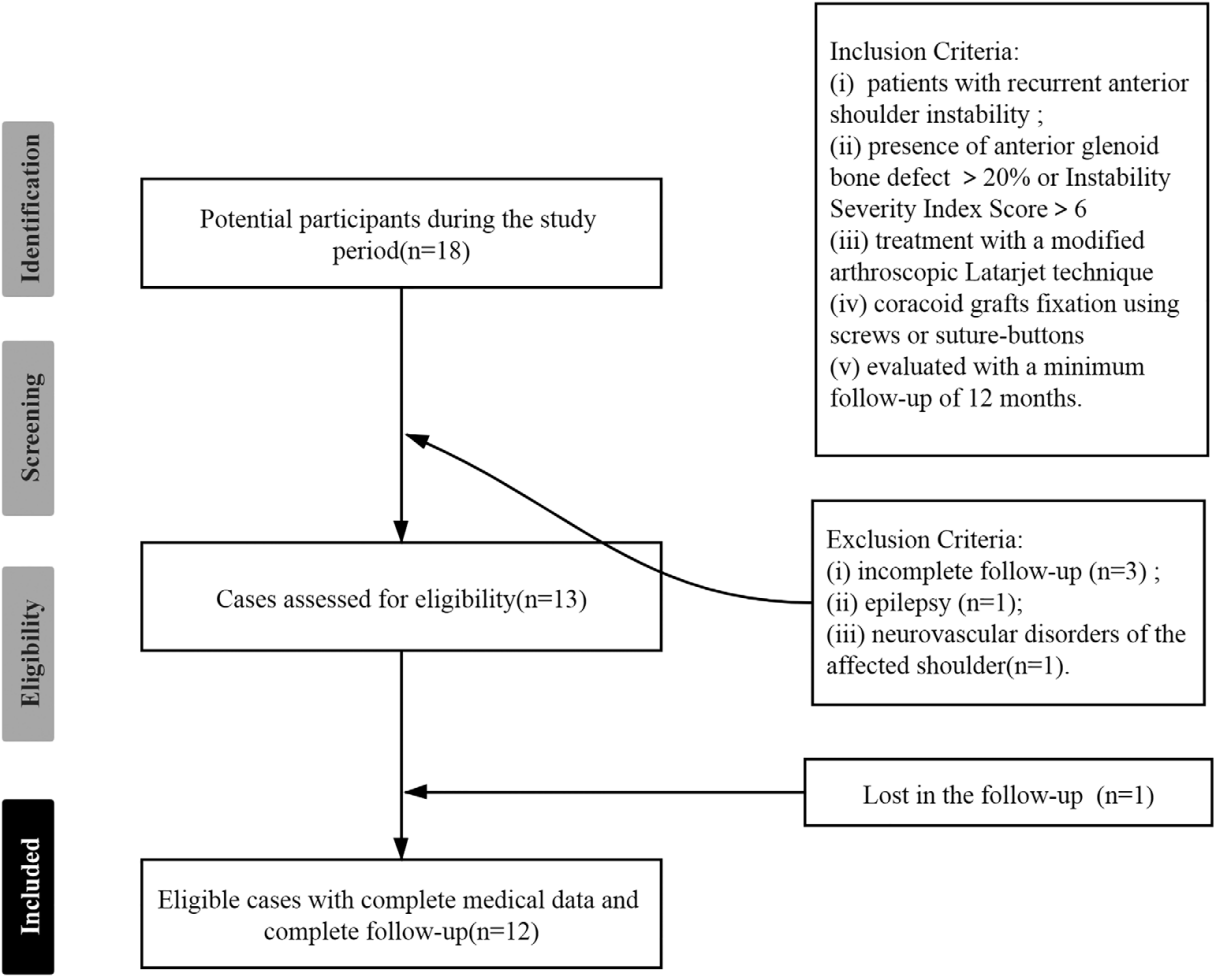
Patients selection in this study.

Exclusion criteria: (i) incomplete follow‐up or loss to follow‐up data; (ii) epilepsy; (iii) neurovascular disorders of the affected shoulder (Fig. 1). The glenoid bone defect was calculated using a bare area method^27^ on the en face view of three‐dimensional (3D) computed tomography (CT). CT data were analyzed using Materialis™ Mimics 19.0 software.

A total of 18 patients who underwent Latarjet procedure between January 2015 and December 2018 were evaluated for inclusion in this study. Among them, three patients missed complete follow‐up and three patients underwent conventional open Latarjet procedures. The final study group included the remaining 12 male patients with a mean follow‐up of 24.9 months (range, 13–53 months). Six patients used screw fixation (Group 1, *n* = 6), another group of patients used suture‐button fixation (Group 2, *n* = 6).

Data of the patient's history, including age, gender, side of affected arm, BMI, and the number of dislocations since fist dislocation were collected.

#### *Grouping*

A total of 12 patients was divided into two groups: screw fixation group and button fixation group. Six patients in screw fixation group underwent arthroscopic Latarjet with screw fixation. Another six patients in screw fixation group underwent arthroscopic Latarjet with button fixation.

### *Surgical Technique*

**Fig 2:**
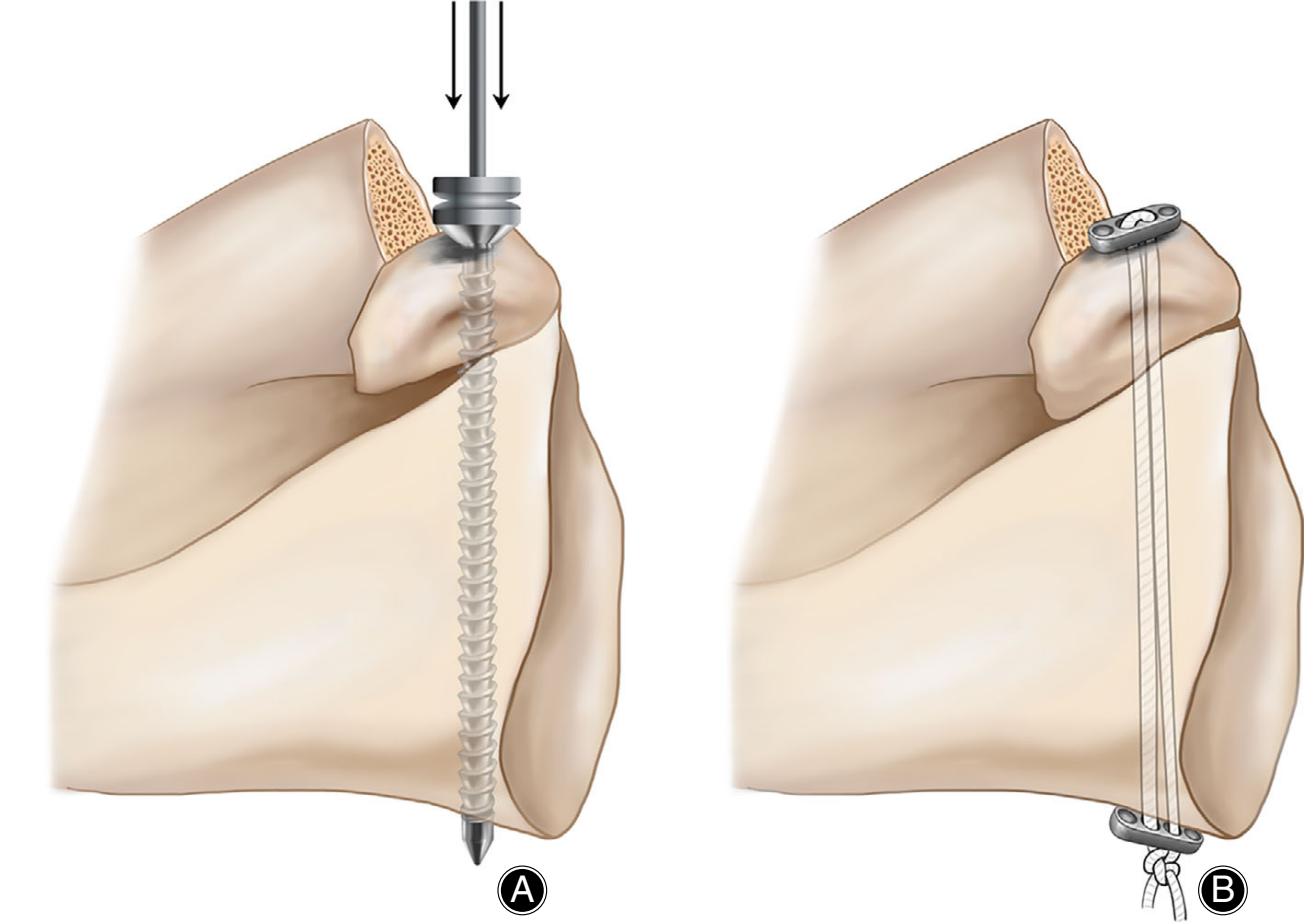
Screw Fixation and Suture‐Button Fixation for the coracoid graft (A, B). Screw fixation of transferred coracoid graft (A). Suture‐Button fixation of transferred coracoid graft (B).

All arthroscopic Latarjet cases were performed by the same senior surgeon (Dr. Zi‐min Wang) at our institution after the patients were given general anesthesia with interscalene block. Patients were placed in beach chair position. The surgical procedures were described below (Fig. 2).

#### *Screw Fixation*

A modified Lafosse's technique by Chun‐yan Jiang *et al*.^28^ was adopted for the screw fixation with a few changes in the management of the anterior capsule and subscapularis split.

#### *Suture‐Button* *Fixation*

**Fig 3:**
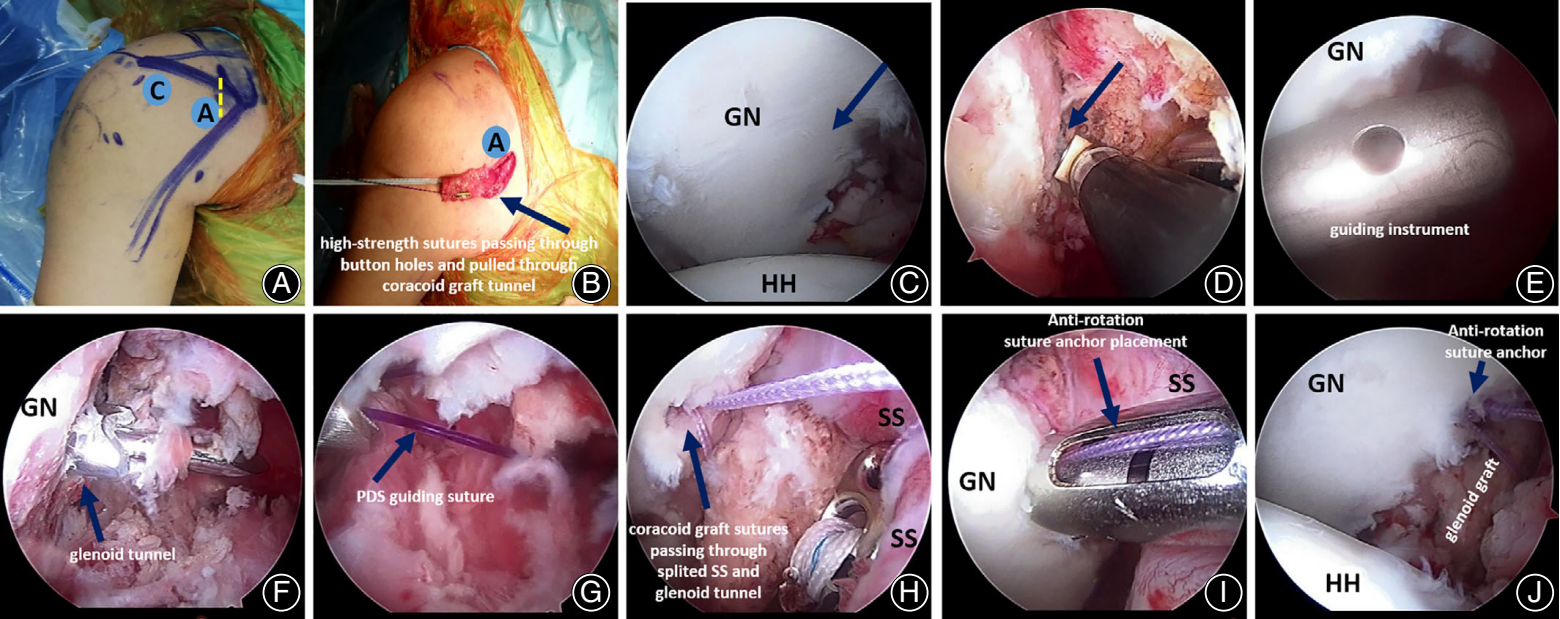
Arthroscopic Latarjet procedure with button fixation. (A, B) The anterosuperior portal (A with black color) which is left by the partly closed incision after coracoid graft harvesting. The standard anterolateral portal was marked on the skin(C with black color). An incision measuring 2.5 cm was made which began from 0.5 cm lateral and 0.5 cm inferior to the coracoid process (yellow dot‐dashed line). An osteotomy of the coracoid process was performed, two bone tunnels were made in the graft along its axis. High‐strength sutures were pulled into the central hole of a suture button and then pulled together to the proximal bone tunnel. (C) Glenoid bone defect was noted from standard posterior portal. (D) Glenoid was marked at the 3 O'clock position through the anterolateral portal. (E‐G) Glenoid tunnel was made under the guidance of a customized instrument, leaving a PDS loop in the tunnel standby. (H) Coracoid sutures passing through splitted SS and then through the glenoid tunnel. (I) A suture anchor was fixed to the glenoid to prevent rotation of the bone graft. (J) The coracoid graft was fixed to the glenoid. GN, glenoid; HH, humeral head; SS, subscapularis.

This surgical procedure consisted of one mini‐open coracoid grafting procedure and other arthroscopic steps^29^ (Fig. 3).

For the mini‐open coracoid grafting procedure, a 2.5 cm skin incision was made along the anterior axillary line, which began from 0.5 cm lateral and 1 cm inferior to the coracoid process. The coracoid process was well exposed. The coracoacromial ligament and partial pectoralis minor were detached from the border of the bone. An osteotomy of the coracoid process was done using an oscillating saw in order to make a bone graft >20 mm long. With a distance of 6 mm, we drilled two bone tunnels with 2.5 mm K‐wire in the bone graft along its axis. The inferior tunnel was just below the bisector of the graft. After decortication of the bone graft, a suture button with three high‐strength sutures in central holes was pulled into the inferior bone tunnel. An isolate high‐strength suture was passed through the superior buttonhole and then pulled into the superior bone tunnel and another suture were passed through conjoint tendon at its attachment on coracoid, respectively. These two sutures could be used as traction sutures while pulling the graft though split subscapularis window. The skin incision was partially closed with 5 mm remaining as the anterior portal, which was exactly located in the anterosuperior side of the subscapularis.

The rest of the steps were performed arthroscopically. Three portals were used, in addition with the anterior coracoid harvested portal, the standard posterior and anterolateral portals were established. Through a standard posterior portal, the intra‐articular viewing was finished to check the long head of biceps tendon (LHBT), the labrum tear “Bankart or Superior Labrum Anterior Posterior (SLAP) lesions” and humeral head injury “Hill–Sachs lesion”, etc. In two cases, the combined SLAP lesions were repaired with suture anchors. The glenoid bone defect was observed and anterior bone bed was decorticated. The glenoid was marked at the 4‐o'clock position. With the help of a customized guiding instrument^29^, a 4 mm diameter tunnel was drilled through the glenoid, leaving a standby Polydioxanon (PDS) loop in the tunnel for suture passing. After careful identification and protection of the axillary nerves with a switch stick, the inferior 1/3 and superior 2/3 of the subscapularis is split horizontally. The coracoid bone graft was pulled into the joint with its sutures guided by the PDS loop and firmly adhered to the glenoid. The posterior button was fixed on the glenoid by the three loop sutures with sliding‐knocking knot. A knotless anchor was fixed at 3 o'clock to prevent coracoid block rotation. Finally, the graft position was checked and portal incisions were closed.

### *Outcome Measures*

Patients in the two groups underwent standardized assessments before surgery, the first day right after surgery, 6 months after surgery, and at final follow‐up (2 years or more) after surgery. The clinical data listed below were collected and measured.

#### *Range of Motion (ROM)*

Active external rotation and active internal rotation at 90° of abduction as well as active elevation were evaluated with a goniometer.

#### *Function Scores*

Shoulder function and stability were evaluated based on the Walch–Duplay score^30^, The American Shoulder and Elbow Surgeons Shoulder (ASES) score, and modified Rowe score^31^.

#### *Visual Analogue Scale (VAS)*

The Visual Analogue Scale (VAS) measures pain intensity. The VAS consists of a 10 cm line, with two end points representing 0 (“no pain”) and 10 (“pain as bad as it could possibly be”)^32^.

#### *American Shoulder and Elbow Surgeons Shoulder (ASES) Score*

The American Shoulder and Elbow Surgeon's Score (ASES) score is a mixed outcome reporting measure, applicable for use in all patients with shoulder pathology regardless of their specific diagnosis. The ASES score contains a physician‐rated and patient‐rated section; however, only the VAS and 10 functional questions are typically used to tabulate the reported ASES score. The total score ‐ 100 maximum points ‐ is weighted 50% for pain and 50% for function^33^.

#### *Rowe Score*

The Rowe score is used to assess the shoulder instability. It is a three‐item physician completed instrument. Its questions address the categories of shoulder stability, motion, and function. Scores range from 0 to 100 and are classified as excellent (90–100 points), good (75– 89 points), fair (50–74 points), or poor (under 50).

#### *Walch–Duplay* *Score*

Walch–Duplay score is the most currently used score in Europe for the assessment of the patient undergoing shoulder stabilization surgery. It is composed of four items: activity, stability, pain, and mobility. Results are classified as excellent (91–100 points), good (76–90 points), fair (51–75 points), or poor (under 50).

### *Radiological Assessments*

CT scans were offered at postoperative day 1, 3 months, 6 months, and 1 year. The position of the transferred coracoid graft and the orientation of the fixation materials were assessed^16^. The ideal position was defined as below the glenoid equator (in the vertical plane) and flush to the glenoid rim (in the horizontal plane)^34^, ^35^. Graft healing was assessed with the same radiologic method according to Lu *et al*.^29^.

Post‐operative CT data of all patients were collected for 3D‐image reconstruction (Materialis™ Mimics 19.0 software) in order to analyze the bony graft volume. Bone block osteolysis of transferred graft was analyzed according to Haeni *et al*.^36^ and described as “partial” around the superior or the inferior fixation, and “total” concerning the entire graft^37^.

### *Statistical Analysis*

Continuous variables are presented as Means ± SD. We compared the pre‐ and postoperative clinical outcomes (shoulder scores and shoulder range of motion) using the matched‐pairs *t*‐test. Post‐operative clinical outcomes (shoulder scores and shoulder range of motion) between screw group and button group were compared using independent two‐sample *t*‐test. Bony graft volume data among groups were compared using one‐way ANOVA test. The statistical analysis was performed using IBM SPSS Statistics 22.0 (IBM Corp, Armonk, NY, USA). *P* value <0.05 was considered statistically significant.

## Results

### *Demographic Data*

Mean follow‐up duration was 24.9 months (range, 10 to 53 months). Six patients with screw fixation and six patients with suture‐button fixation were included in this study. The 12 patients were all men with six right shoulders and six left shoulders. The average age at the time of surgery was 23.2 ± 6.2 years (range, 17 to 34 years) in screw fixation group while 23.2 ± 4.6 years (range, 19 to 31 years) in suture‐button fixation group. Mean BMI was 23.4 ± 2.0 kg/m^2^ in screw fixation group and 24.9 ± 4.7 kg/m^2^ in suture‐button fixation group. The number of dislocations from first dislocation to surgery was 11.3 ± 2.5 in screw fixation group (two patients were not included) and 8.5 ± 5.2 in suture‐button fixation group (two patients were not included). Two patients in screw fixation group could not recall the accurate number of dislocations but the number of dislocation was more than 20 times, while the number of dislocations of two patients in suture‐button fixation group was more than 30 times.

### *Function Score*

The VAS score, ASES score, Rowe score and Walch–Duplay score of 12 patients before surgery and at final follow‐up were available in Table 1. The difference between preoperative and postoperative VAS score, ASES score, Rowe score and Walch–Duplay score was significant in each group (*P*<0.01). But there was no significant difference between two groups in postoperative VAS score, ASES score, Rowe score, and Walch–Duplay score.

**TABLE 1:** Function results exhibited preoperatively and at final follow‐up

Indexes	Screw fixation			Suture‐button fixation		
	Pre‐op	Post‐op	*P* value	Pre‐op	Post‐op	*P* value
VAS	2.7 ± 0.5	1.2 ± 0.8	0.017	3.0 ± 0.9	1.5 ± 1.0	0.003
Range of motion (°)						
Forward flexion	175.0 ± 8.4	178.3 ± 4.1	0.465	174.8 ± 10.2	175.0 ± 5.5	0.975
External rotation at 90° of abduction	77.5 ± 5.2	71.7 ± 4.1	0.034	72.5 ± 6.9	68.3 ± 7.5	0.093
Internal rotation at 90° of abduction	66.7 ± 6.1	67.5 ± 6.1	0.611	68.3 ± 11.3	66.7 ± 7.5	0.576
ASES score	68.9 ± 7.9	91.1 ± 6.1	0.005	68.9 ± 8.9	87.5 ± 6.7	<0.001
Rowe score	25.0 ± 8.4	92.5 ± 4.2	<0.001	21.7 ± 13.7	93.3 ± 4.1	<0.001
Walch‐Duplay score	12.5 ± 15.1	91.7 ± 4.1	<0.001	18.3 ± 20.7	88.3 ± 7.5	<0.001

Data were reported as mean ± SD unless otherwise indicated. ASES, American Shoulder and Elbow Surgeons Shoulder Score; VAS, visual analog scale

#### *VAS* *Score*

The average VAS score improved from 2.7 ± 0.5 preoperatively to 1.2 ± 0.8 postoperatively in screw fixation group and 3.0 ± 0.9 preoperatively to1.5 ± 1.0 postoperatively in suture‐button fixation group.

#### *ASES* *Score*

The average ASES score improved from 68.9 ± 7.9 preoperatively to 91.1 ± 6.1 postoperatively in screw fixation group and 68.9 ± 8.9 preoperatively to 87.5 ± 6.7 postoperatively in suture‐button group.

#### *Rowe Score*

The average Rowe score improved from 25.0 ± 8.4 preoperatively to 92.5 ± 4.2 postoperatively in screw fixation group, and 21.7 ± 13.7 preoperatively to 93.3 ± 4.1 postoperatively in suture‐button fixation group.

#### *Walch–Duplay* *Score*

The average Walch–Duplay score improved from 12.5 ± 15.1 preoperatively to 91.7 ± 4.1 postoperatively in screw fixation group and 18.3 ± 20.7 preoperatively to 88.3 ± 7.5 postoperatively in suture‐button fixation group.

### *Range of Motion*

Range of motion was measured before surgery and at final follow‐up. The forward flexion, active external rotation and active internal rotation of 12 patients were available in Table 1. The forward flexion was 175.0° ± 8.4° preoperatively and 178.3° ± 4.1° postoperatively in screw fixation group while 174.8° ± 10.2° preoperatively and 175.0° ± 5.5° postoperatively in suture‐button fixation group. The average of active external rotation was 77.5° ± 5.2° preoperatively and 71.7° ± 4.1° postoperatively in screw fixation group while 72.5° ± 6.9° preoperatively and 68.3° ± 7.5° postoperatively in suture‐button fixation group. The average of active internal rotation was 66.7° ± 6.1° preoperatively and 67.5° ± 6.1° postoperatively in screw fixation group while 68.3° ± 11.3° preoperatively and 66.7° ± 7.5° postoperatively in suture‐button fixation group. There were no significant differences in the range of motions between the two groups.

### *Coracoid Graft Positioning*

**Fig 4:**
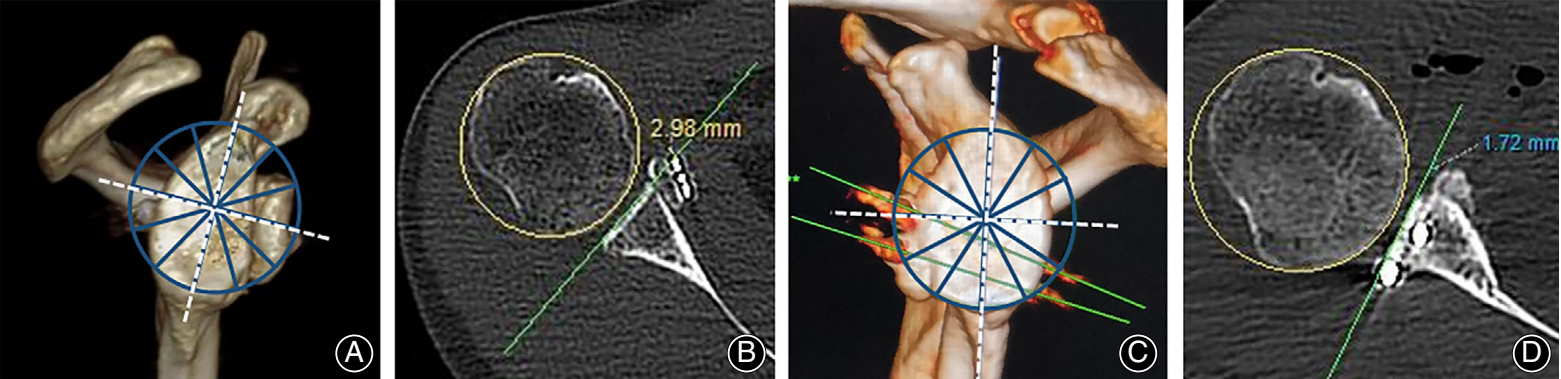
(A) En face view showing the graft positioned at the equator level in the suture‐button fixation group. (C) En face view showing the graft positioned under the equator level in the screw fixation group. Axial view showing that the graft was fixed too laterally in suture‐button fixation group (B) and medially in screw fixation group (D). The equator of the glenoid was defined as a line perpendicular to and bifurcating the line connecting the most superior–inferior aspect of the glenoid.

The data of the CT scan assessment for graft positioning are demonstrated in **Table** **2**. Overall, 83.3% of the coracoid grafts were placed congruent with the glenoid articular surface line, with only two grafts exhibiting slight medial placement in screw fixation group (33.3%) and one graft exhibiting slight lateral placement in suture‐button fixation group(16.7%) (Fig. 4). From the en face view, 66.7% of all grafts were placed under the equator with only two grafts positioned at the equator level in suture‐button fixation group (33.3%), one graft positioned over the equator (16.7%), and one at the equator level (16.7%), respectively, in screw fixation group. No secondary rotation of the graft was found.

**TABLE 2:** Coracoid graft position in relation to the glenoid evaluated on CT scan performed postoperatively at day 1

Coracoid graft position	Screw fixation (*n* = 6)		Button fixation (*n* = 6)	
	No. of shoulders	%	No. of shoulders	%
Horizontal position				
Too medial (>5 mm medial to the glenoid rim)	2	33.3	0	0
Too lateral (>5 mm lateral to the glenoid rim)	0	0	1	16.7
Flush (correct graft position)	4	66.7	5	83.3
Vertical position				
Over the equator (>50% of bone block over equator line)	1	16.7	0	0
At the equator (>25% of bone block over equator line)	1	16.7	2	33.3
Under the equator (correct graft position)	4	66.7	4	66.7

### *Coracoid Graft Healing*

**Fig 5:**
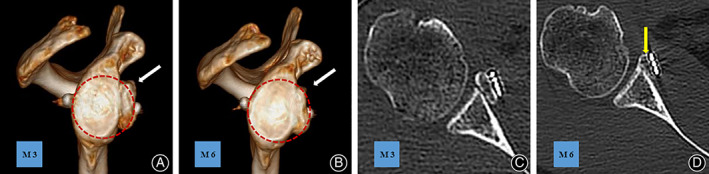
Remolding and healing of the coracoid graft in the Suture‐button fixation group. From the en face view (A, B), the glenoid and graft were fused and remodeled as an intact body, with partial bony absorption occurred outside of the “best‐fit” circle (white arrow). From the axial view (C, D), the glenoid and graft were remodeled to a congruent concavity with the ipsilateral humeral head (yellow arrow) 6 months postoperatively. M3, 3 months postoperative follow‐up; M6, 6 months postoperative follow‐up.

Based on the CT scan assessment performed 6 months after surgery, the graft exhibited perfect union in all patients at 6 months follow‐up (Fig. 5). The glenoid and graft were fused and remodeled analogous to the shape of the intact glenoid. Grafts showed from an en face view that bone absorption occurred outside of the “best‐fit” circle^38^. From the axial view 6 months postoperatively, the glenoid and graft were remodeled to a congruent concavity with the ipsilateral humeral head. Partial graft osteolysis was found in two patients. Only one patient in screw fixation group exhibited total graft osteolysis at 2 years follow‐up.

**Fig 6:**
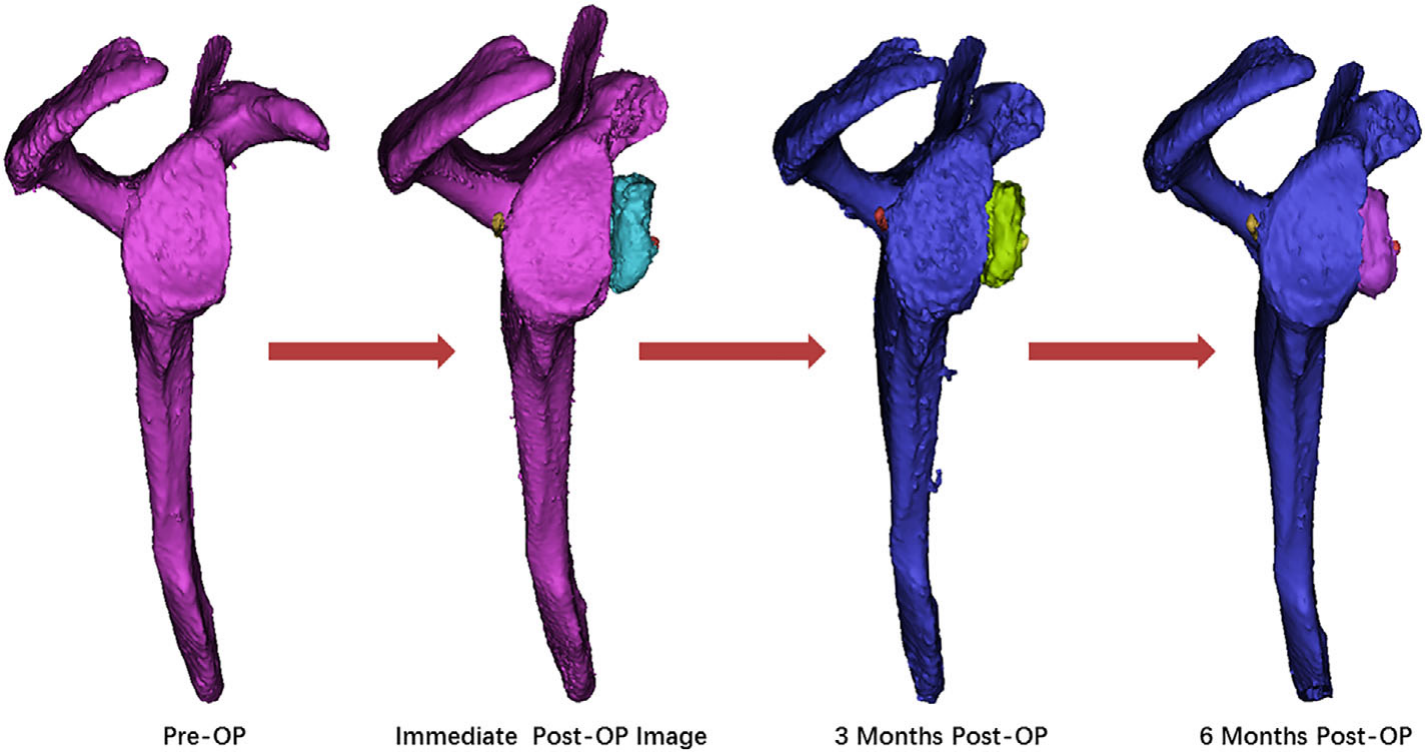
Post‐operative results of bony‐graft volume (Mimics 19.0 Software). 3D image reconstruction analysis demonstrated that bony grafts volume kept on decreasing during the reconstructing and fusing phases of bone healing in Suture‐button fixation group.

Three‐dimensional image reconstruction analysis demonstrated that bony grafts volume kept on decreasing along with the reconstruction and fusion phases (Fig. 6). The average bony absorption rates of the coracoid grafts were 13.82% and 5.26% in screw fixation group and suture‐button fixation group, respectively, at 3 months postoperative follow‐up, and 25.2% and 10.18%, respectively, at 6 months postoperative follow‐up (Table 3) .

**TABLE 3:** Post‐operative follow‐up of coracoid graft absorption rate (Mimics 19.0 Software, %)

	M0	M3	M6
1	0.00	10.14	21.52
2	0.00	4.05	19.71
3	0.00	19.65	32.38
4	0.00	18.69	29.01
5	0.00	14.37	24.01
6	0.00	13.06	21.74
Mean	0.00	13.82	25.20
7	0.00	6.38	11.51
8	0.00	4.80	9.62
9	0.00	5.08	9.32
10	0.00	5.16	10.31
11	0.00	5.67	11.36
12	0.00	4.74	9.50
Mean	0.00	5.26	10.18

M0, immediate post‐operative testing; M3, 3 months' post‐operative follow‐up; M6, 6 months' post‐operative follow‐up.

### *Orientation of the Fixation Devices*

**Fig 7:**
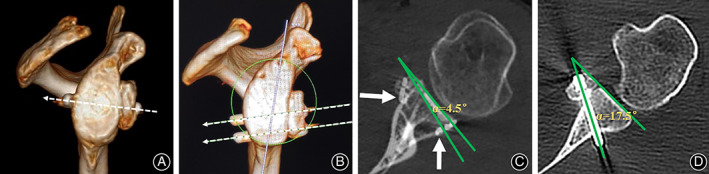
Mechanical stress direction of the coracoid graft: (A) Arthroscopic Latarjet technique with suture‐button fixation; (B) Arthroscopic Latarjet technique with screw fixation. Angulation of fixation devices relative to joint line in axial plane; (C) Arthroscopic Latarjet technique with suture‐button fixation (white arrows pointed to the two suture buttons based on picture overlap technique); (D) Arthroscopic Latarjet technique with screw fixation. The angulation was evaluated on the axial CT scan by measuring the angle (α) between the axis of the fixation device and the glenoid fossa.

Based on the axial 2D CT scan image, the orientation of the fixation device was 19.6° ± 9.9° in the screw fixation group compared with 15.3° ± 6.0° in the button fixation group (Fig. 7C, D). From the en face view on the 3D CT reconstruction image, the mechanical stress direction in fixing the coracoid graft was from the anteroinferior to the post‐superior in suture‐button fixation (Fig. 7A) and from anterosuperior to the post‐inferior in screw fixation (Fig. 7B).

### *Risk Factors for Graft Osteolysis*

**Fig 8:**
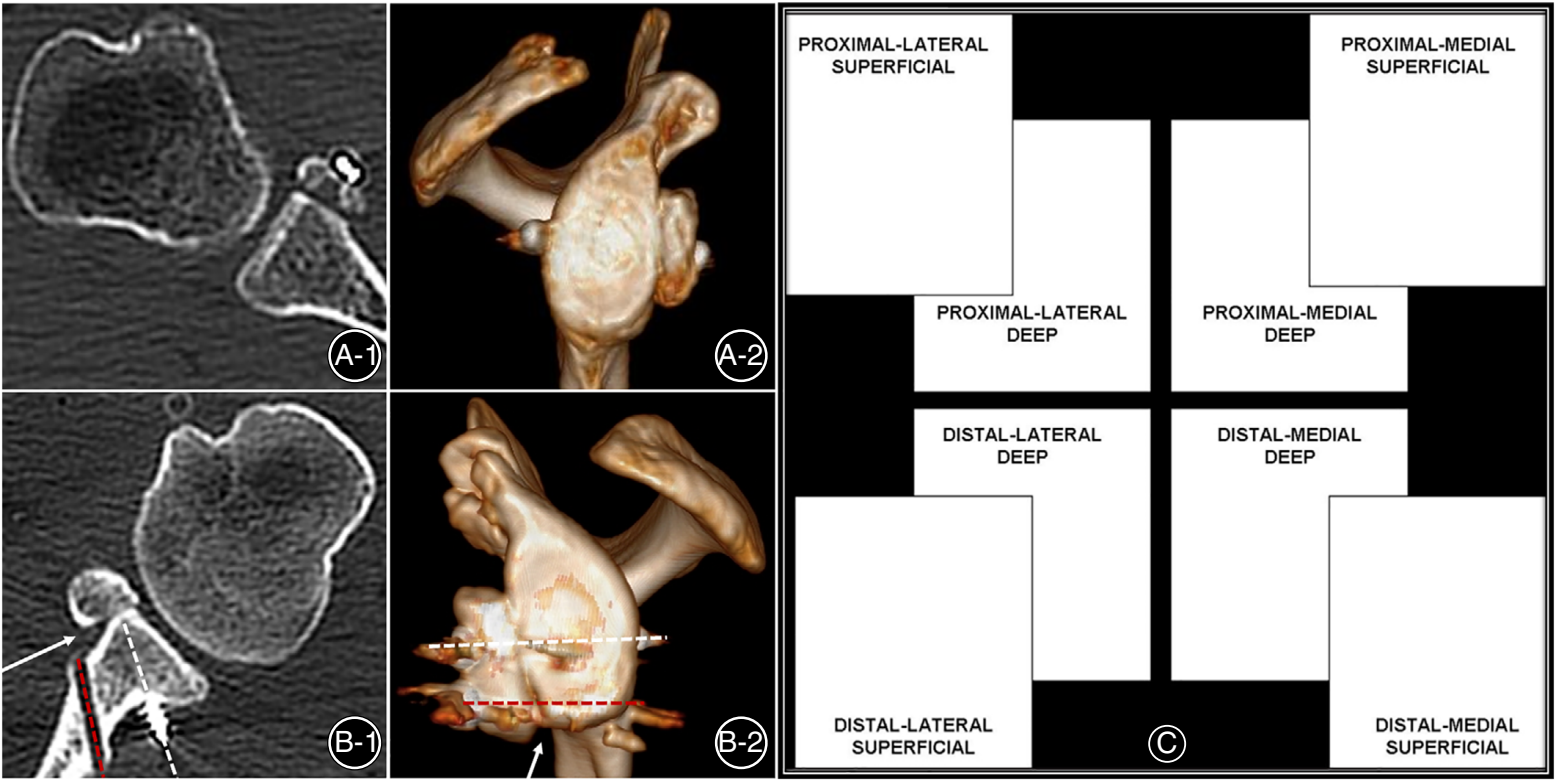
Coracoid bone graft contact. (A1‐A2) complete contact between the coracoid bone graft and the glenoid rim on both the axial view and en face view using suture‐button fixation; (B1‐B2) the medial part of the proximal coracoid bone graft could not be closely attached to the anterior surface of the glenoid rim using screw fixation, leaving an obvious gap (white arrow) between the graft and glenoid rim(with dotted lines pointed to the fixing screws). (C) diagram of partitioning in 8 parts of the coracoid bone graft in right shoulder (frontal view)^36^.

We found that the medial part of the proximal coracoid bone graft^39^ could not be closely attached to the anteroinferior surface of the glenoid rim using screw fixation technique (Fig. 8). In suture‐button fixation group, no obvious gap between the coracoid bone graft and the anteroinferior surface of the glenoid rim was found.

### *Complications*

No severe complication was found in all patients during the follow‐up.

## Discussion

Open Latarjet has been proven a successful and effective procedure for recurrent anterior shoulder instability in patients with glenoid bone loss ≥20%^40^. This technique was modified by Patte *et al*.^41^ since it was first introduced by Latarjet in 1954^42^. Laffose was the first who initiated this technique arthroscopically without changing the method of fixing the coracoid graft with screws^11^. Recently, Boileau *et al*.^13^ introduced a novel arthroscopic suture‐button fixation method that provides more choices for surgeons when performing Latarjet surgery.

### *Screws and Suture‐Button in Latarjet Procedure*

Arthroscopic Latarjet procedure is a challenging yet viable technique to treat anterior shoulder instability, achieving equal results to the open technique with advantages of the arthroscopic setting^43^. Several complications such as graft osteolysis, non‐union, and mal‐positioning regarding this technique have been reported previously^44^, ^45^. Jiang *et al*.^16^, ^46^ found that patients in the arthroscopic Latarjet group showed notably less graft resorption compared with patients in the open Latarjet group 1 year after surgery. Of all the patients, Jiang *et al*. used screws in fixing the coracoid graft. Nicolas *et al*.^47^ reported that Latarjet procedure with suture‐button fixation exhibited a low complication rate. In a biomechanical study, Kazum *et al*.^48^ found no significant differences in the maximal load‐to‐failure between suture‐button fixation and screw fixation. But the most common failure mode of the endo‐button was glenoid bone fracture while the screw was graft fracture. Boileau *et al*.^23^ showed mid‐term results of suture‐button arthroscopic Latarjet with a low instability recurrence rate and excellent return to pre‐injury activity level, and considered it as a safe and reliable alternative to screw fixation. Lu *et al*.^13^ modified Boileau's method^13^ and found perfect graft healing as well as remodeling results in arthroscopic Latarjet procedure with suture‐button fixation. Dalmas *et al*.^49^ reported a union rate of 64% at 3 months and 93% at 1 year with double‐button fixation arthroscopic Latarjet procedure.

### *Grafts Healing with Screw Fixation and Suture‐Button Fixation*

In this study, we found all patients achieved bone union. Two patients exhibited slight graft osteolysis postoperatively (one patient in screw fixation group categorized as Grade I according to Jiang *et al*.^28^, one patient in button fixation group with bony resorption occurred outside of the “best‐fit” circle). Only one patient in the screw fixation group showed total graft osteolysis with almost a bare screw exposed in the fixing area 2 years after surgery. However, the patient had no obvious discomfort during shoulder movement with only slight limitation on external rotation. We have not found any relative risk factors contributing to osteolysis in this study. The mechanical stress directions in fixing the coracoid grafts were observed from the en face view of 3D CT images, but no specific findings were achieved. Whether fixed from the anteroinferior to the posterosuperior or from other directions, the bony grafts tended to heal properly. It depended on the experience and preference of the surgeon when choosing the “right” direction to fix a bony graft during the operation. Based on the postoperative CT data, we analyzed the bony grafts volume within 6 months. Our 3D image reconstruction analysis confirmed that bony grafts volume tended to decrease as time went by. Screw fixation group exhibited more obvious osteolysis when compared with suture‐button fixation group. To the best of our knowledge, we are the first to evaluate bony graft healing and osteolysis in arthroscopic Latarjet clinical study using Mimics software.

The coracoid graft exhibited perfect union and remodeled to a congruent concavity with the ipsilateral humeral head in button group even when slight resorption was noted 3 months after surgery. Contrary to our findings, research by Nazmiet *et al*. showed that lesser stress magnitudes were observed with endo‐button fixation method compared with screw and wedge plate fixation which is important for graft osteolysis^50^. Lu *et al*.^29^ recently reported that even the grafts fixed too laterally presented a phenomenon of remodeling and became flush with the glenoid rim over time. They believed button fixation is flexible and might help relieve impingement of the humeral head caused by hardware above the glenoid level. In our study, we found the similar phenomenon, that bone graft positioned laterally exhibited excellent remodeling and fusion to the glenoid in button fixation group. Considering that there is a certain visual bias in arthroscopic surgery, we believe that this fixation method has certain fault tolerance and is more friendly to surgeons.

From clinical and biomechanical standpoints^8^, ^51^, ^52^, overhanging graft positioning has been associated with an increased risk for secondary osteoarthritic changes. It's believed that medial positioning of up to 4 mm with regard to the articular cartilage is acceptable, whereas a lateral to overhang of more than 1 mm is probably not^34^, ^51^, ^52^. In our study, only two grafts exhibited slightly medial placement in screw fixation group (33.3%) and one graft exhibited slight lateral placement in button fixation group (16.7%). All grafts showed bone union and remodeling, except one patient in screw fixation group exhibited total osteolysis without any clinical symptoms at 2‐years follow‐up. Neyton *et al*.^53^ evaluated graft position and fixation in 208 patients and found that bone block positioning in the arthroscopic screw group was significantly more lateral compared with the open Latarjet and the bone block in the arthroscopic button group was also more lateral compared with the open Latarjet but flush with the join line at 0 mm. Whether performed arthroscopically or openly, it's difficult to place the coracoid graft at an ideal position during the operation. How to avoid postoperative bone/joint injury and promote bone healing have become our main concerns. Even with slight lateralization, the graft showed fairly excellent radiographic healing in suture‐button fixation group whereas the only one case exhibiting total osteolysis in screw fixation that might render moderate to severe osteoarthritis in long‐term follow‐up. With this in mind, we would prefer button fixation in our future cases.

We found that 16.7% of grafts in screw fixation group positioned over the equator compared with 0% incidence observed in the suture‐button fixation group. Most grafts were positioned at the equator or under the equator level (91.7%). The optimum position of the coracoid graft is controversial. Nourissat *et al*.^54^ reported in a biomechanical study that the better location of the bone block was at the 4 o'clock position. Laffose *et al*.^12^ indicated that the ideal placement is between 3 and 5 o'clock. Boileau *et al*.^14^ described that the bone block should be positioned below the equator of the native glenoid. Jiang *et al*.^16^ determined that the center of the graft below the equator of the glenoid was the optimum superior–inferior position of the coracoid. Technically, there's a 2D vision viewing bias during arthroscopic surgery and the level of the subscapularis split may also have an effect on the intraoperative position of the graft. Jiang *et al*.^16^ speculated that the bulkier lower subscapularis muscle caused by the high split made it difficult to place the graft in the optimum position (between 3 and 5 o'clock). In the present study, no severe complications were observed in both groups even when the graft malposition occurred immediately after fixation intraoperatively. Using a customized drilling device during arthroscopic Latarjet procedure with button fixation, we could achieve a better tunnel position that made the bone block location between 3 and 5 o'clock compared with screw fixation method.

Excessive screw obliquity may cause impingement with the humeral head, promoting osteoarthritis of the glenohumeral joint^13^. The results of our study showed that the orientation of the fixation device was 19.6° ± 9.9° in the screw fixation group compared with 15.3° ± 6.0° in the button fixation group (*P* < 0.0001). It was easier to achieve a better tunnel position parallel to the joint line using suture‐button fixation technique during surgery. Furthermore, a very medial “M portal” is needed in order to make the screws parallel to the articular surface of the glenoid using screw fixation technique which might cause severe axillary nerve injury or cosmetic concern in female patients. When compared with screw fixation, button fixation technique does not need an extra “M portal,” thus greatly reducing the risk of neurovascular damage during arthroscopic anterior manipulation.

Many factors are associated with osteolysis. Di Giacomo *et al*.^39^ demonstrated that solely increasing compression was not enough to achieve less coracoid bone graft osteolysis by comparing screw fixation with a more rigid mini‐plate fixation in Latarjet procedure. They divided the coracoid bone graft into eight parts when analyzing the osteolysis of the coracoid grafts (Fig. 7C) and found the most relevant osteolysis was represented by the superficial part of the proximal coracoid^39^. A finite element analysis showed that all fixation types produced higher von Mises stress (VMs) around the implant and on the superior part of the coracoid graft but the VMs distribution resulting from single endo‐button fixation was the least and caused less osteolysis^50^. The findings in our study were correlated with the results of those articles, we found that the medial part of the proximal coracoid bone graft could not be closely attached to the anteroinferior surface of the glenoid rim in screw fixation group but not in suture‐button fixation group, which means there were excessive compressive stresses between the superior part of the coracoid and the glenoid rim using screw fixation. We believed that the suture‐button fixation helped ensure better bone contact between the coracoid bone graft and the glenoid with less compressive stress.

### *Limitations*

There are some limitations to this study. First, errors may occur during the radiographic assessments. These measurements were analyzed by a single observer in our study, lacking intra‐observer repeatability test. Second, the number of cases enrolled was small. Data from a larger sample size is needed in the future. Third, the postoperative follow‐up time in this study is short. A medium‐ and long‐term follow‐up study with more patients included will be needed in the future.

## Conclusions

To the best of our knowledge, this is the first study to analyze coracoid graft healing and osteolysis in arthroscopic Latarjet procedure using Mimics 3D image reconstruction method. Our radiographic assessments demonstrated that the coracoid bony grafts healed to the glenoid perfectly with less osteolysis in suture‐button fixation when compared with screw fixation.

Both suture‐button and screw fixation techniques in arthroscopic Latarjet procedure revealed excellent clinical outcomes with low complication rates in the early follow‐up. The suture‐button technique exhibited a flexible fixation pattern that allowed for self‐correction to some extent, even slight lateralization could finally remodel over time. However, more accurate results require controlled studies with more clinical cases followed over longer periods of time.
